# Extracellular Enzyme Activity and Its Implications for Organic Matter Cycling in Northern Chinese Marginal Seas

**DOI:** 10.3389/fmicb.2019.02137

**Published:** 2019-09-13

**Authors:** Yi Li, Lin-Lin Sun, Yuan-Yuan Sun, Qian-Qian Cha, Chun-Yang Li, Dian-Li Zhao, Xiao-Yan Song, Min Wang, Andrew McMinn, Xiu-Lan Chen, Yu-Zhong Zhang, Qi-Long Qin

**Affiliations:** ^1^State Key Laboratory of Microbial Technology, Marine Biotechnology Research Center, Shandong University, Qingdao, China; ^2^College of Marine Life Sciences, Ocean University of China, Qingdao, China; ^3^Laboratory for Marine Biology and Biotechnology, Qingdao National Laboratory for Marine Science and Technology, Qingdao, China; ^4^Institute for Marine and Antarctic Studies, University of Tasmania, Hobart, TAS, Australia

**Keywords:** extracellular enzyme, distribution pattern, enzyme-producing clades, DOC utilization, environmental factors, Chinese marginal seas

## Abstract

Extracellular enzymes, initiating the degradation of organic macromolecules, are important functional components of marine ecosystems. Measuring *in situ* seawater extracellular enzyme activity (EEA) can provide fundamental information for understanding the biogeochemical cycling of organic matter in the ocean. Here we investigate the patterns of EEA and the major factors affecting the seawater EEA of Chinese marginal seas. The geographic distribution of EEA along a latitudinal transect was examined and found to be associated with dissolved organic carbon. Compared with offshore waters, inshore waters had higher enzyme activity. All the tested substrates were hydrolyzed at different rates and phosphatase, β-glucosidase and protease contributed greatly to summed hydrolysis rates. For any particular enzyme activity, the contribution of dissolved to total EEA was strongly heterogenous between stations. Comparisons of hydrolysis rates of the polymers and their corresponding oligomers suggest that molecule size does not necessarily limit the turnover of marine organic matter. In addition, several typical enzyme-producing clades, such as Bacteroidetes, Planctomycetes, Chloroflexi, *Roseobacter*, *Alteromonas*, and *Pseudoalteromonas*, were detected in the *in situ* environments. These enzyme-producing clades may be responsible for the production of different enzymes. Overall, each enzyme was found to flexibly respond to environmental conditions and were linked to microbial community composition. It is likely that this activity will profoundly affect organic matter cycling in the Chinese marginal seas.

## Introduction

Microorganisms play a key role in organic matter remineralization in the ocean. An estimated 50% of primary production in the surface water is transformed, repackaged, and respired via the microbial loop (Azam et al., 1983; Arnosti et al., 2005; Azam and Malfatti, 2007). Most of the organic matter in the ocean is in the form of chemically complex macromolecules and these polymers are too large to be transported across the cytoplasmic membrane (Arnosti, 2004). They must initially be hydrolyzed into small molecules (<600 Da) by extracellular enzymes before uptake by the microbial cell (Weiss et al., 1991). Extracellular enzymes are, therefore, key factors affecting the organic matter cycling in marine ecosystems (Arnosti, 2010; Orsi et al., 2018).

Marine microbial extracellular enzymes are either cell-associated or dissolved in the water column (Chróst, 1991; Baltar et al., 2010). Cell-associated enzymes are bound to the cell surface, or localized in the periplasmic space (Reintjes et al., 2019). These enzymes present a cost-efficient strategy for free-living microbes. Due to the dilute nature of dissolved organic matter (DOM), they can help the cell to preferentially access DOM (Chróst, 1991). However, the substrate must penetrate the cell wall or be physically located near the cell (Allison et al., 2012). Some polysaccharides substrates could be directly taken up into the periplasm of ‘selfish’ organism without production of extracellular hydrolysis products (Reintjes et al., 2017, 2019; Hehemann et al., 2019). Dissolved enzymes, which belong to a kind of “living dead” realm (Baltar, 2018), may originate from active secretion by cell (Alderkamp et al., 2007), bacterial starvation (Albertson et al., 1990) and changes in cell permeability (Chróst, 1991). Besides, they can also be produced in the process of grazing on bacterial communities and release during viral lysis (Chróst, 1991; Bochdansky et al., 1995; Baltar, 2018). As they spread, these enzymes can hydrolyze distant substrates, but hydrolysis products may not be harvested by the parent cell. Due to long lifetime, especially in the deep waters, dissolved enzymes perform their important function away from the cell (Baltar et al., 2013). In many cases, dissolved EEA could make up a substantial proportion (up to 100%) of the total marine EEA (Baltar et al., 2010, 2016; Arnosti, 2010), which could indicate a disconnection between marine microbes and enzymatic activities (D’ambrosio et al., 2014). For particle-associated microbes, a ‘secreting dissolved enzyme’ strategy may be profitable due to the high nutrient concentrations on particles or relaxing requirement for cellular contact with particle organic matter (Vetter and Deming, 1999; Grossart et al., 2007; Baltar et al., 2019). These microbes exhibit a loose hydrolysis-uptake coupling with the substrate (Baltar et al., 2010). Sometimes, the ‘secreting dissolved enzyme’ strategy may also be favorable for free-living microbes through cooperative efforts, although it presumably is costly (Pai et al., 2012; Celiker and Gore, 2013).

Because most marine microbes cannot be cultured and because of the uncertainty of genomic and *in situ* gene investigations, there is no adequate information that either represents extracellular enzyme activity (EEA) in marine ecosystems or element recycling on global scales (Arnosti, 2010). Thus, *in situ* measuring of EEA that can provide a fundamental understanding of the biogeochemical cycling of organic matter is important and has been recognized in field studies (Arnosti, 2010). However, most measurements of enzyme activity use low molecular weight substrate proxies to extrapolate biopolymer enzyme activity. These proxies lack the three-dimensional structure of biopolymers in solution and cannot reflect the real degradation of polymers (Arnosti, 2010). Hence, little is known about the hydrolysis rates of real polymers. A further limitation is that the substrate proxies do not represent the activity of endo-acting enzymes that cleave to the inside of a polymer chain. Although several fluoresceinamine labeled polysaccharides have been used to measure polysaccharide degrading enzymes in the marine ecosystem (Reintjes et al., 2017, 2019), the activity of extracellular enzymes on proteinaceous polymers, that constitute a large proportion of organic matters of primary production, has not been measured. Furthermore, almost nothing is known about the differences in hydrolysis rates between polymers and their corresponding oligomers.

The southern Yellow Sea is a semi-enclosed marginal sea bordered by the Chinese mainland and the Korean Peninsula. In the south, it is open to the East China Sea, which is one of the largest marginal seas in the world and has an extensive continental shelf area (Lee et al., 2014; Wei et al., 2016).

Freshwater discharge from the Yangtze River mixes with the surrounding high salinity waters in the East China Sea (Su and Weng, 1994). Due to high riverine nutrient load as well as pollution, phytoplankton blooms often occur in the southern Yellow Sea and East China Sea region (Fan and Song, 2014). Little is known about the distribution of EEA and the key factors affecting enzyme activity in this region. One limitation of measuring *in situ* EEA is that the natural concentration of extracellular enzymes is often too low to be measured directly. In this study, a tangential flow filtration system was used to concentrate the extracellular enzymes so that the EEA on biopolymer substrates (carboxymethyl cellulose (CMC), chitin, alginic acid and casein) could be measured directly. Based on the same chemical structure, we compared the hydrolysis rates of the polymers and their corresponding oligomers to study whether substrate size can affect the hydrolysis rate. The pattern of dissolved and total EEA and corresponding determinant environmental factors were also analyzed.

## Materials and Methods

### Sampling and Physicochemical Analysis

**FIGURE 1:**
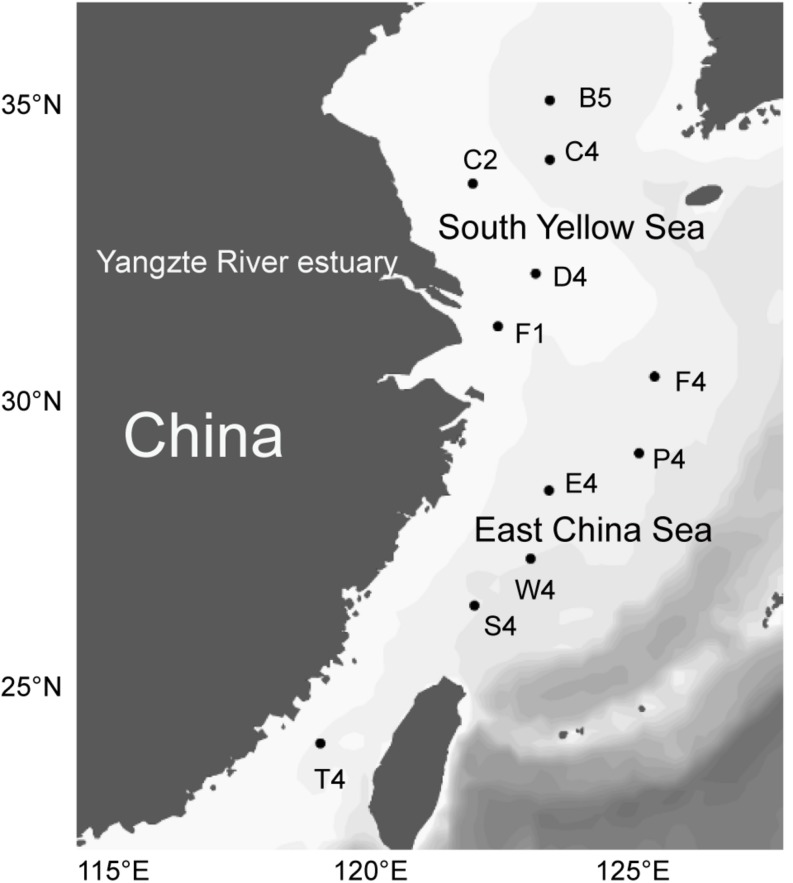
Sampling stations in the southern Yellow Sea and East China Sea.

A total of 11 samples were sampled from two regions, i.e., the inshore region with a bottom depth <55 m (including stations C2, D4, and F1) and the offshore region with a bottom depth >55 m (including stations B5, C4, F6, P6, S4, E4, W4 and T4). Surface water was taken at approximately 2 m water depth using a submersible pump during a cruise of R/V Dong Fang Hong-2, from 26 June to 19 July, 2018 (Figure 1). After prefiltration through 20-μm-pore-size filters (Millipore Co., United States) to eliminate large particles and organisms, 10-l seawater samples were filtered on board through 0.22-μm polycarbonate membranes (Millipore Co., United States). Filters for DNA extraction were immediately placed in liquid nitrogen and frozen at −80°C (Thermo Scientific, United States). Additional pre-filtered 10-l seawater samples were concentrated to 50 ml using a tangential flow filtration system with 5000-Dalton hollow modified polyethersulfone membranes (Spectrum Laboratories, Inc., United States). The concentration process was completed within 2 h and the samples were used for EEA measurement. For this, 25 ml of the concentrated seawater samples were gently and manually filtered through a 0.22-μm-pore-size polypropylene Millex-GP Syringe filter (Millipore Co., United States) to separate cell-associated enzymes from dissolved enzymes following a previously reported protocol (Kim et al., 2007). The water samples for enzyme activity measurement were stored at 4°C until the experiments began within 1 h in the onboard lab. Water temperature, salinity, depth, pH and dissolved oxygen were recorded by the onboard conductivity-temperature-depth sensor (Sealogger, Sea-Bird Co., United States). Water samples for DOC and chlorophyll a (chl-a) were immediately frozen at −20°C. Water samples for nutrient contents (NH_4_^+^, NO_2_^–^, NO_3_^–^, and PO_4_^3–^) were filtered through 0.45-μm cellulose acetate membranes and then measured by spectrophotometric and colorimetric analyses. Chl-a concentration was extracted with 90% acetone and then determined by using a spectrophotofluorimetry method (Holm-Hansen et al., 1965). DOC content was measured by a TOC analyzer (Thermo Flash 2000 Elemental Analyzer, United States).

### Measurements of Enzyme Activity

The hydrolysis rates of nine different substrates (five low molecular weight4-nitrophenyl analogs and four high molecular weight (HMW) polymers) were measured. Enzyme assays were conducted at 25°C (average ambient temperature). To study the effect of warming of seawater on enzymatic activity, enzyme assays were also conducted at 35°C (high temperature), which represents a “heat wave” type of scenario. The measured EEA in the unfiltered seawater was defined as the total enzyme activity and the measured EEA of the seawater passing through the 0.22-μm filter was defined as the dissolved enzyme activity. In this study, they were measured with high substrate concentrations (well above *in situ* concentrations) to ensure EEA be measured as much as possible. Thus, the measured enzyme activities are potential values, which are not indicative of the actual rates of enzymatically catalyzed reactions under natural conditions (Wallenstein and Weintraub, 2008).

The hydrolysis rates of 4-nitrophenyl laurate (C12), 4-nitrophenyl phosphate disodium salt hexahydrate (PDSH), 4-nitrophenyl-α-D-glucopyranoside (AG), 4-nitrophenyl-β-D- glucopyranoside (BG) and 4-nitrophenyl-N-acetyl-β-D-glucopyranoside (NAG) were measured to estimate the activities of lipase, phosphatase (Pase), α-, β-glucosidase (AGase, BGase) and N-acetyl-glucosaminidase (NAGase), respectively. Three polysaccharides (CMC, chitin and alginic acid) were used as substrates to measure the activities of cellulase, chitinase, and alginate lyase, respectively. Casein was used as the substrate to measure protease activity. All chemicals were obtained from Sigma-Aldrich (United States).

After the test, all reaction systems and incubation time were determined to ensure the reasonability of data. For 4-nitrophenyl analogs, the reaction system contained 30 μl 50 mM substrate and 470 μl of the concentrated seawater sample. After incubation for 5 h, the reaction was terminated by Tris-NaOH (pH 12.0) and the absorbance of the reaction mixture was measured at 405 nm. For the hydrolysis of the CMC and chitin, 30 μl of 0.5 mg/ml CMC or 0.1% (w/v) colloidal chitin and 270 μl of the water sample were mixed. The colloidal chitin was prepared as previously described (Wietz et al., 2015). The reaction was terminated by NaOH and the contents of the produced reducing sugar was determined using the 3-Methyl-2-benzothiazolinono-hydrazone method (Sastry and Vijaya, 1987). The casein reaction system contained 50 μl 2% (w/v) casein and 450 μl of the water sample; the hydrolysis rate was measured as previously reported (Chen et al., 2002). Finally, tyrosine contents were measured by absorbance at 660 nm. The alginic acid reaction system contained 50 μl of 4 mg/ml alginic acid and 450 μl of the water sample. After the termination of the reaction, the absorbance of the reaction mixture was measured at 235 nm. All HMW substrate reactions were incubated for 20 h. This long-time incubation may cause the overestimate of the EEA in the unfiltered seawater as microbes may grow and secrete new enzymes. Autoclaved seawater was used as the sample control; from this, the abiotic rate of hydrolysis was determined and subtracted for each assay. Triplicate incubations were conducted with unfiltered and filtrate water for each station. A standard curve was produced to allow conversion of absorbance into product concentration using a known amount of the corresponding monomer of the substrate except for alginic acid. One unit (U) of alginate lyase activity was defined as the amount of enzyme needed to produce an *Absorbance* 235 increase of 0.001 per hour (Xu et al., 2017). The sum of the hydrolysis rates measured for each station was calculated. Besides, the overall contribution of dissolved EEA to total EEA for the tested substrates was analyzed.

### DNA Extraction, PCR Amplification, and Phylogenetic Analysis

Total genomic DNA from each sample was extracted following the protocol of PowerWater^®^ DNA Isolation Kit (QIAGEN, Germany). Amplification of bacterial 16S rRNA gene fragments was performed using barcoded sequencing primers 338F (5′-ACTCCTACGGGAGGCAGCA-3′) and 806R (5′-GGACTACHVGGGTWTCTAAT-3′). Sequencing was conducted using an Illumina MiSeq platform (Majorbio Bio-Pharm Technology Co., Ltd., China). Raw reads were trimmed, merged and filtered using Usearch (Edgar, 2018). Obtained reads were clustered into OTUs at a 97% identity, and finally an OTUs table was generated. Taxonomy was assigned against RDP Naive Bayesian rRNA Classifier at an 80% confidence threshold based on representative OTU sequences (Liu and Wong, 2013). The phylogeny was estimated using the neighbor-joining algorithm based on the V3-V4 region of bacterial 16S rRNA gene sequences in MEGA 7 (Stecher et al., 2016). The phylogenetic tree was constituted of 31 OTU sequences and 16 referenced sequences from the NCBI database. Bacterial clades were defined by the marker sequences from published phylogenies.

### Statistical Analysis

The cluster diagram, non-metric multidimensional scaling (NMDS) and one-way analysis of similarity (ANOSIM) were conducted using Primer 6 (Plymouth Marine Laboratory, United Kingdom). Cluster diagrams of total EEA and dissolved EEA were also performed on data from two different temperature regimes. Bacterial community compositions and comparisons to relative abundances in different stations were performed by R software. Redundancy analyses (RDAs), with Monte Carlo permutation tests, were implemented to estimate the relationships between total or dissolved EEAs and environmental factors by CANOCO (Version 5, Microcomputer Power). Screened environmental factors without multicollinearity effects (variance inflation factor <20) were used to explain enzyme activity variability. A Pearson’s correlation index was calculated to determine correlations between DOC content and latitude. Wilcoxon’s rank test was performed using R software to compare different enzyme activities from the inshore and offshore regions. The Illumina sequences were deposited in the National Center for Biotechnology Information (NCBI) Short Read Archive database under accession number PRJNA517539.

## Results

### Physicochemical Characterization

A total of 11 water samples from the southern Yellow Sea and East China Sea were obtained from a depth of approximately 2 m (Figure 1). Environmental variables of all the seawater samplings are listed in Supplementary Table S1. The temperature at the sampling stations ranged from 19.2 to 28.2°C with an average of 25.3°C. Stations D4 and F1 had very low salinity (<3.0%), which was almost certainly related to the input of freshwater from the Yangtze River. Inorganic nutrient concentrations (PO_4_^3–^, NO_2_^–^, NO_3_^–^ and NH_4_^+^) were significantly higher in the inshore regions than those in the offshore regions (Wilcoxon test, *p* < 0.05). The chl-a content ranged from 0.16 to 3.80 μg L^–1^ and was significantly higher in the inshore regions than that in the offshore regions (Wilcoxon test, *p* < 0.05). It also showed strong positive correlations with NO_2_^–^ (*r* = 0.80, *p* < 0.01), NO_3_^–^ (*r* = 0.79, *p* < 0.01) and NH_4_^+^ (*r* = 0.96, *p* < 0.01). The NDMS and cluster dendrogram analyses showed that the inshore and the offshore stations clustered into two different groups based on differences in physicochemical factors (Supplementary Figure S1) (ANOSIM, *r* = 0.97, *p* = 0.006).

### Patterns of Extracellular Enzyme Activities

**FIGURE 2:**
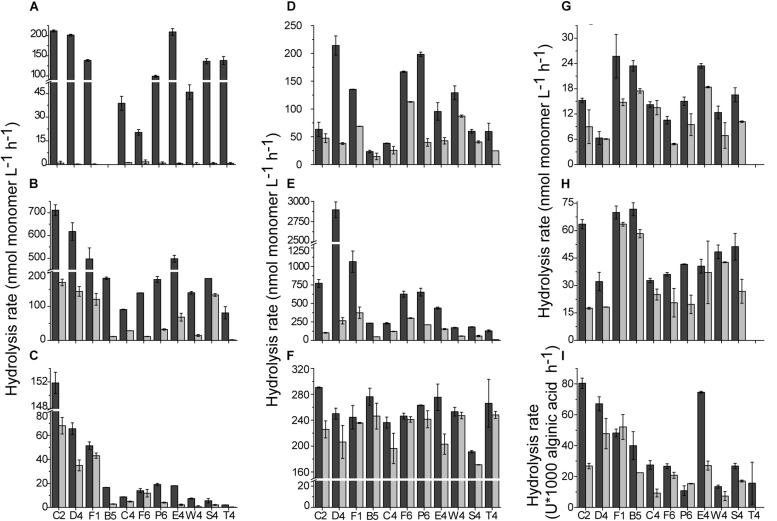
The hydrolysis rates of tested substrates at 35°C. **(A)** α-D-glucopyranoside (AG). **(B)** β-D-glucopyranoside (BG). **(C)** N-acetyl-β-D-glucopyranoside (NAG). **(D)** Laurate (C12). **(E)** Phosphate disodium salt hexahydrate (PDSH). **(F)** Casein. **(G)** Chitin. **(H)** Carboxymethyl cellulose (CMC). **(I)** Alginic acid. Values are the mean hydrolysis rate in unfiltered water (dark gray), and 0.22-μm filtered water (light gray) at each station.

For unfiltered and filtered concentrated water sample from each station, the enzyme assays were conducted both at 35 and 25°C, respectively. In the 35°C assays, hydrolysis of all tested substrates was observed (Figure 2). For total EEAs, among small molecule sugars, the average hydrolysis rate of BG (302 ± 120 nmol L^–1^ h^–1^) was significantly higher than that of NAG (32 ± 12 nmol L^–1^ h^–1^) (*p* < 0.01) and AG (124 ± 48 nmol L^–1^ h^–1^). For the polysaccharides, the average hydrolysis rate of CMC was 49 ± 14 nmol L^–1^ h^–1^, which was higher than that of chitin (16 ± 6 nmol L^–1^ h^–1^). The average activity of alginate lyase was 40 ± 25 × 10^–3^ U/L. Compared with the hydrolysis rates of polysaccharides CMC and chitin, the hydrolysis rate of casein was much higher (254 ± 25 nmol L^–1^ h^–1^) (*p* < 0.01). The hydrolysis rate of C12 ranged from 23 nmol L^–1^ h^–1^ to 214 nmol L^–1^ h^–1^. PDSH was hydrolyzed with the highest rate (2895 nmol L^–1^ h^–1^) among all tested substrates. Dissolved EEAs were lower than total EEAs for all tested substrates. Though AG was hydrolyzed rapidly by the total AGases, there was scarcely detectable hydrolysis in the filtered water, which suggests that most of the AGases in the coastal waters were cell-associated. In contrast, the hydrolysis of casein, CMC, chitin and alginic acid was predominantly driven by dissolved extracellular enzymes.

In the 25°C assays, the hydrolysis rates of eight substrates were measured due to scarcely detectable hydrolysis of AG (Supplementary Figure S2). Hydrolysis of all tested substrates at 25°C also showed an obvious rate variation along stations. Hydrolysis rates of eight substrates in total and dissolved waters ranged from nearly 790 nmol L^–1^ h^–1^ (for PDSH at station F1) to undetectable (for AG at station B5 and CMC and chitin at station T4). The Q_10_ value (represents the factor by which the hydrolysis rate increases from 25 to 35°C) for each enzyme (except for AGase) from the different stations was ranged from 1.1 to 2.9, showing that different enzyme classes have different Q_10_ values and the stoichiometry of organic matter remineralization would change as the ocean warms. Therein, for total and dissolved EEAs, Q_10_ values of BGase were the highest (2.2 and 2.9) among the eight tested enzymes.

### Comparison of Summed Enzyme Activities

**FIGURE 3:**
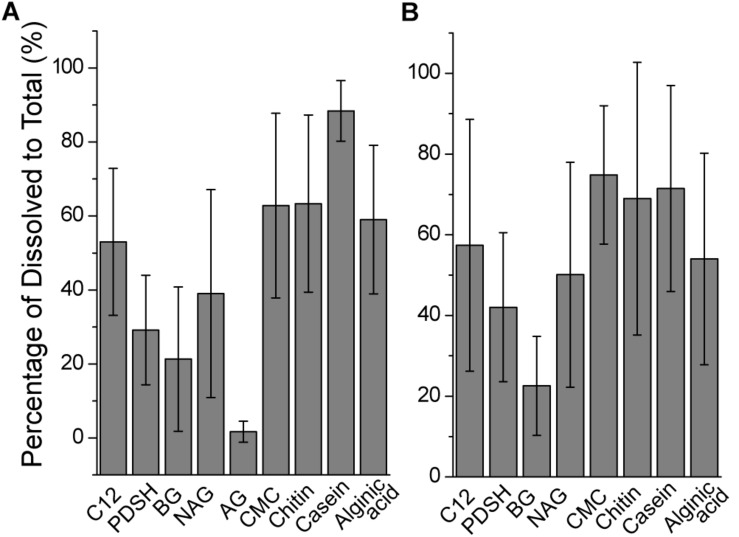
The contributions of dissolved to total extracellular enzyme activities across stations. **(A)** At 35°C. **(B)** At 25°C.

The contribution of the dissolved EEA to the total EEA was analyzed (Figure 3). The contribution of the dissolved AGase activity to the total AGase activity was extremely low (<2%). Small proportions of Pase, BGase and NAGase activity were also found in the filtered water, which suggests that these enzymes were mainly cell-associated. In contrast to carbohydrase degrading small molecule sugars, polysaccharase were mainly dissolved. During the incubations at 35°C, the contributions of the dissolved to the total cellulase, chitinase and alginate lyase activity were 62 ± 25%, 63 ± 24% and 59 ± 20%, respectively (Figure 3A). During the incubations at 25°C, for cellulose, the contribution of the dissolved to the total activity was the highest (75 ± 17%), followed by chitinase (69 ± 34%) and alginate lyase (54 ± 26%) (Figure 3B). Protease activity in the filtered water made a large contribution (88 ± 8% at 35°C; 71 ± 25% at 25°C). The contribution of the dissolved fraction to the total activity of all tested enzyme activity varied significantly. This may be partly explained by microbial survival strategies, available substrate concentrations (Traving et al., 2015) and the method used to measure enzyme activity (Arnosti, 2010).

**FIGURE 4:**
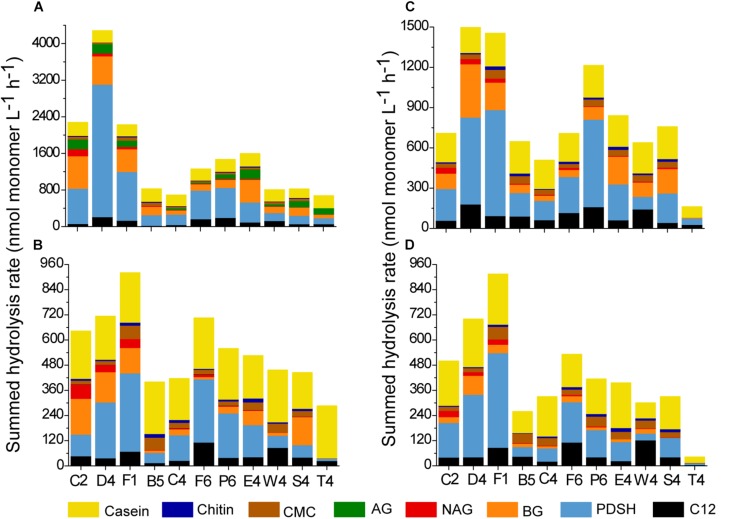
Summed hydrolysis rates at each station. **(A)** The unfiltered waters at 35°C. **(B)** The 0.22-μm filtered waters at 35°C. **(C)** The unfiltered waters at 25°C. **(D)** The 0.22-μm filtered waters at 25°C.

The hydrolysis rate of the different substrates (except for alginic acid) at each station was summed to present the potential hydrolysis at each station (Figure 4). Summed activities of total and dissolved extracellular enzymes varied across stations. For total EEAs, the highest summed enzyme activity was detected at station D4 (4283 nmol L^–1^ h^–1^ at 35°C; 1566 nmol L^–1^ h^–1^ at 25°C). The total enzyme activity at stations C2 and F1 were also high. For dissolved EEAs, the highest summed enzyme activity was detected at station F1 (920 nmol L^–1^ h^–1^ at 35°C; 915 nmol L^–1^ h^–1^ at 25°C), followed by the station D4 (713 nmol L^–1^ h^–1^ at 35°C; 700 nmol L^–1^ h^–1^ at 25°C). For each station, Pase, BGase and protease activity made a major contribution to the summed activity. Inshore waters, especially in the estuary, presented higher enzyme activity than the offshore waters. The cluster dendrograms of enzyme activity showed that the enzyme activity from inshore waters grouped together and were different from those of offshore waters (Supplementary Figure S3).

**FIGURE 5:**
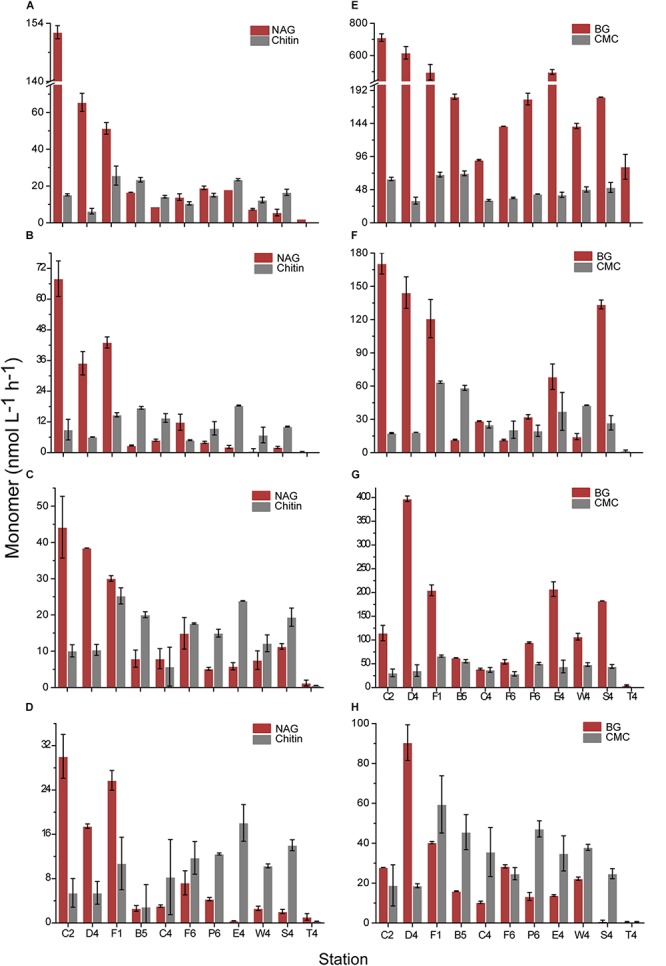
Comparison of hydrolysis rates of polymer and corresponding monomer. **(A,E)** The unfiltered waters at 35°C. **(B,F)** The 0.22-μm filtered waters at 35°C. **(C,G)** The unfiltered waters at 25°C. **(D,H)** The 0.22-μm filtered waters at 25°C. **(A–D)** Present NAG and chitin. **(E–H)** Present BG and CMC.

Based on the same chemical structure and the different degree of polymerization, the hydrolysis rates of the polymers (CMC and chitin) and corresponding oligomers (BG and NAG) were compared to analyze the effect of substrate size on hydrolysis rate (Figure 5). In the unfiltered water, the hydrolysis rates of BG from all stations were significantly higher than those of CMC (*p* < 0.01). NAG in each inshore station was hydrolyzed at a higher rate than chitin, but in each offshore station, it was hydrolyzed at a lower rate. This suggests that some polymers could be hydrolyzed at lower rates in inshore regions but at higher rates in offshore regions compared with corresponding oligomers. This situation also occurred in the dissolved NAGase and chitinase (Supplementary Table S2). For dissolved BGase and cellulase, the BGase activity at most stations at 35°C was higher than the cellulase activity.

### Environmental Factors Associated With Enzyme Activity

**FIGURE 6:**
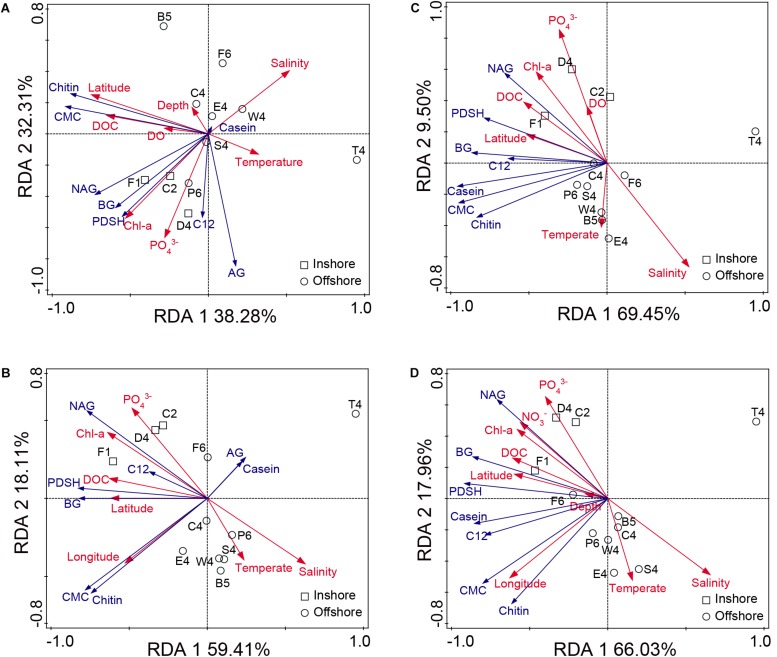
Redundancy analyses (RDA) between environmental parameters and EEAs. **(A)** The total enzyme activities at 35°C. **(B)** The dissolved enzyme activities at 35°C. **(C)** The total enzyme activities at 25°C. **(D)** The dissolved enzyme activities at 25°C.

The RDAs were performed to investigate the environmental factors responsible for shaping enzyme activity (Figure 6). The environmental factors without multicollinearity effects provided high explanatory powers (>70%) for the total and dissolved enzyme activities. For total EEAs at 35°C, Monte Carlo permutation tests showed that latitude was the most significant environmental factor, followed by DOC, chl-a and PO_4_^3–^ (Figure 6A). The hydrolysis rates of CMC and chitin showed close, positive relationships with DOC and latitude, respectively. The hydrolysis rates of NAG, BG and PDSH were found to be positively correlated with chl-a and PO_4_^3–^. For dissolved EEAs at 35°C, DOC was the only significant factor accounting for the variability (*p* < 0.05) (Figure 6B). The hydrolysis rate of NAG was found to be closely related to chl-a and PO_4_^3–^. The hydrolysis rates of BG and PDSH were positively correlated with latitude and DOC, respectively. For EEAs at 25°C, latitude had the highest RDA explanatory power (23.8 and 33.1%) on the total and dissolved enzyme activity, followed by PO_4_^3–^, chl-a, and salinity (Figures 6C,D). The hydrolysis rates of BG, NAG and PDSH were also highly positively related to latitude, DOC and chl-a. These RDA results showed that latitude and DOC were associated with the distribution of EEA on a large scale. Chl-a, salinity and PO_4_^3–^ also were correlated to NAGase, BGase and Pase activity at inshore stations.

## Discussion

### Specific Enzyme Activity Involved in Carbon, Nitrogen and Phosphorus Cycling

Lipases, alginate lyases and carbohydrases play important roles in the marine carbon cycle (Imai et al., 1993; Xu et al., 2017). In the pelagic ocean, lipases from marine gliding bacteria were detected and could decompose cell membranes of dead organisms such as protozoa and phytoplankton (Imai et al., 1993). In this study, *in situ* lipase activity (∼200 nmol L^–1^ h^–1^) was also detected. Alginate lyases, which are synthesized by microbes, brown seaweeds and marine molluscs (Yin, 2015; Xu et al., 2017), showed different activities in the tested stations. Although AGase and BGase are both able to hydrolyze oligosaccharides and release monosaccharides (Dick, 2011), the hydrolysis rate of BG was much higher than that of AG in the unfiltered or 0.22-μm filtered water (Figures 2A,B). This may be due to BGase’s central position in global carbon cycling as BGase catalyzes the final step in the breakdown of cellulose, which mediates the subsequent supply of monomer glucose to microorganisms (Luo et al., 2017).

As an indicator of nitrogen acquisition, NAGase has an important function in nitrogen cycling (Shi et al., 2012; Sistla and Schimel, 2013). The general substrates for NAGase are chitin and peptidoglycan, which both are structural materials in marine invertebrates, fungi, algae and bacteria (Riemann and Azam, 2002). In the inshore stations with high availability of nitrogen, high NAGase activity was detected, which implies a rising microbial demand for nitrogen (Figure 2C and Supplementary Figure S2C) (Min et al., 2011). Protease activity, which acts to degrade multiple proteinaceous substrates that can be utilized by microorganisms as both carbon and nitrogen sources, was detected in all samples.

In many cases, Pase activity generally acts as a good indicator of the phosphorus supply for bacteria and phytoplankton (Hoppe, 2003; Luo et al., 2017). However, in some cases, Pase activities are decoupled from phosphate concentrations (Thomson et al., 2019). In comparison with the other enzymes tested in this study, Pase showed the highest activity at both 35 and 25°C (Figure 2E and Supplementary Figure S2E), the distributions of which were positively correlated with these of PO_4_^3–^ (Pearson’s correlation test, for total EEA at 35°C, *r* = 0.86, *p* < 0.01; for dissolved EEA at 35°C, *r* = 0.41, *p* < 0.05; for total EEA at 25°C, *r* = 0.54, *p* < 0.05; for dissolved EEA at 25°C, *r* = 0.65, *p* < 0.05) (Figure 6). Particularly, at stations D4 and F1 near the Yangtze River estuary, PDSH was hydrolyzed very rapidly. Interestingly, as the end-product, high concentration inorganic phosphorus apparently did not inhibit Pase activities. This may be due to the presence of long lived Pase and the potential for spatial and temporal decoupling between the inorganic phosphorus concentrations and the Pase activity (Thomson et al., 2019). It has been reported that the proportion of the dissolved fraction to the total Pase activity can be as high as 70% (Hoppe, 2003; Thomson et al., 2019). In this study, the dissolved fraction also made a relatively large contribution to the total Pase activity (up to 40%). These dissolved Pase may originate from marine protozoa, bacterial stress and mortality (Baltar et al., 2019). Due to long lifetime, they can persistently function in the ocean, which serve as an example of the potential critical role that dissolved enzymes play in the ecology and biogeochemistry of the ocean (Baltar, 2018; Thomson et al., 2019).

In this study, the hydrolysis rates of different substrates were relatively higher but in the same order of magnitude as compared with previous studies. For example, it was reported that the Pase activities could reach 200 nmol L^–1^ h^–1^ in the coastal southern California (Allison et al., 2012), which was lower than Pase activities measured (300–700 nmol L^–1^ h^–1^) in this study. Enzymatic activities in the open ocean were orders of magnitude lower than that in the marginal seas (Baltar et al., 2010, 2013). This may be due to the input of terrestrial freshwater, which might have provided both high nutrients and microbial cells that could stimulate enzyme production (Arnosti, 2004; Baltar et al., 2010). Besides, human activities, high incubation temperature and different methods of measurement are likely to be responsible for the high EEA in this study. The detected activity of a diverse range of extracellular enzymes indicates that the tested organic substrates, such as chitin, lipid and protein, can be hydrolyzed and utilized by marine microorganisms. Elevated temperature increases the enzymatic activity, implying that warming of seawater could accelerate the organic matter cycling rates in the marine ecosystem.

### Differences in Enzyme Activity Toward Polymer and Corresponding Oligomer

Compared to the polymers CMC and chitin, the oligomers BG and NAG from inshore waters, especially in the estuary, were hydrolyzed at higher rates than those from offshore waters, which indicates that the hydrolysis rates of the smaller substrates were susceptible to terrestrial influences. Frequently, hydrolysis rates of polymers are lower than those of corresponding oligomers; this was found for CMC and BG at most stations. However, there were inconsistencies in that HMW substrates were hydrolyzed at higher rates (Pantoja and Lee, 1999) or were hydrolyzed at the same rates as their smaller components (Arnosti and Repeta, 1994; Arnosti et al., 1994). In offshore stations, for example, chitin was hydrolyzed faster than NAG (Figures 5A–D). Molecule size may not necessarily limit the turnover of organic matter in marine systems (Arnosti, 2014). The nature of the substrate structure is also important to determine the hydrolysis rate (Pantoja and Lee, 1999). The hydrolysis rates of these substrates were also related to action modes of enzymes. In aquatic ecosystems, polymers are efficiently cleaved into oligomers by endohydrolases or monomers by exohydrolases (Obayashi and Suzuki, 2005; Weiner et al., 2008). However, consumption of these polymers may also occur through the production of oligomers that are of a suitable size for bacterial uptake, not just monomers (Arnosti et al., 2014). Some microbes even prefer to take up oligomers relative to monomers (Cotta and Zeltwanger, 1995).

### Effects of Environmental Factors on Enzyme Activity

Different kinds of enzymes are known to responded flexibly to changes in environmental conditions and this causes spatial and temporal variation in their distribution in the ocean (Arnosti, 2014). Near the Yangtze River estuary, the stations D4 and F1 had low salinities (<3.0%), due to input of substantial freshwater from Yangtze River. The freshwater input might have provided a terrestrial source of nutrients or microbial cells that could stimulate enzyme production (Allison et al., 2012; Millar et al., 2015), as observed at stations D4 and F1, which had high inorganic nutrient concentrations (PO_4_^3–^, NO_2_^–^, NO_3_^–^, and NH_4_^+^). Phytoplankton dynamics, especially blooms accompanied by changes in nutrient conditions and CO_2_, can affect marine enzyme activity distribution (Allison et al., 2012; Arnosti, 2014). In line with this, elevated enzyme activity was found in the inshore stations with high chl-a contents (Figure 5).

**FIGURE 7:**
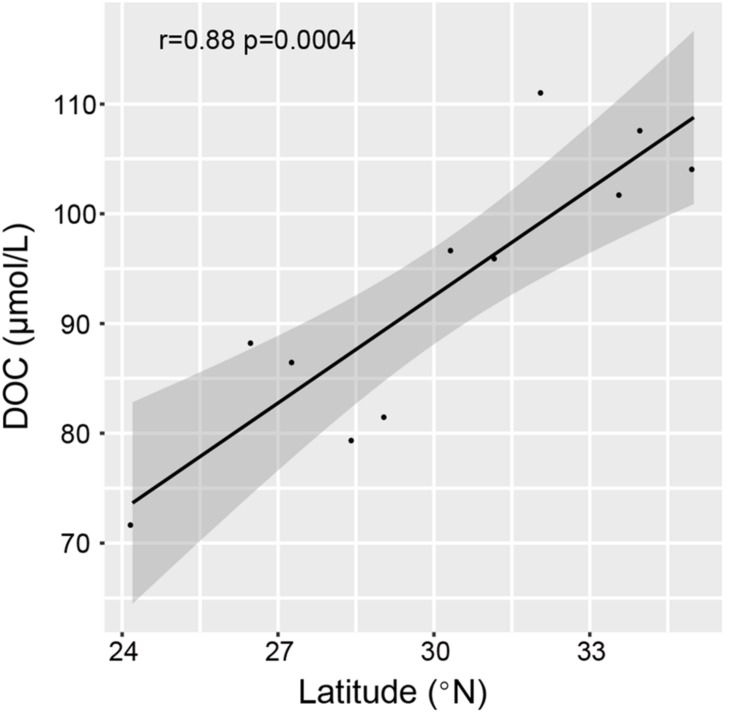
The correlation between DOC contents and latitude among stations as shown by the Pearson correlation.

Based on the RDA analyses, latitude and DOC were closely associated with the total and dissolved enzyme activity (Figure 6). Summed hydrolysis rates for all tested substrates increased first and then decreased with increasing latitude (Supplementary Figure S4). However, latitude alone is a geographical factor and cannot have a direct relationship with the distribution of enzyme activity. It was found that DOC had a high collinearity with latitude (Figure 6), and DOC was significantly positively related to latitude through the Pearson’s correlation test (*r* = 0.88, *p* < 0.01) (Figure 7). Therefore, it is speculated that DOC was an important factor associated with the geographic distribution of EEA. As one of the largest active organic carbon reservoirs on earth, marine DOC is the basic component of the matter cycle and shapes the variation of microbial EEA in the ocean (Arnosti, 2010; Li et al., 2018).

In this study, for each tested substrate, the hydrolysis rate at 35°C was higher than that at 25°C. As reported, without the effect of other environment factors, increases in temperature alone should, within limits, result in increased enzyme activity (Price and Sowers, 2004). Except for the direct effect of temperature on EEA, however, warming is also expected to affect oceanic enzyme activity on a large scale by changing vertical mixing and nutrient distribution patterns in these water bodies (Pomeroy and Wiebe, 2001). This might cause a shift in the spectrum of EEA acting at different ocean levels (Cunha et al., 2010). Interestingly, different Q_10_ values were found for different enzyme classes in this study, which implied that stoichiometry of organic matter remineralization would change as the ocean warms.

### Connecting Bacterial Communities and Enzyme Activity

**FIGURE 8:**
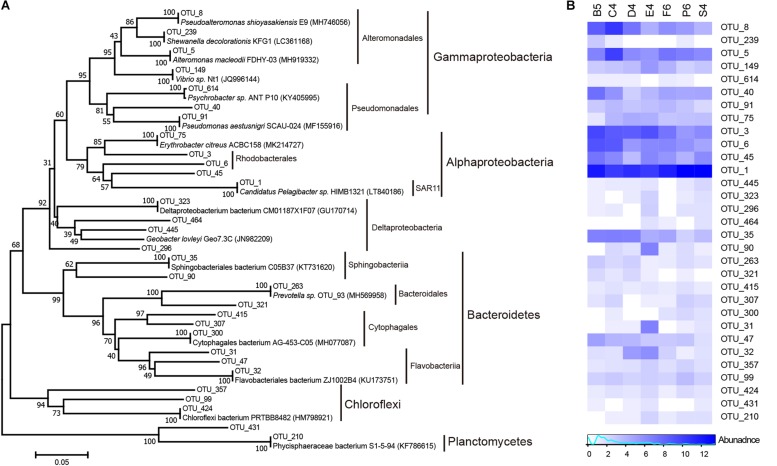
Major producing enzyme clades in the north Chinese marginal seas. **(A)** Neighbor-joining phylogeny of bacterial phylotypes, based on partial 16S rRNA sequences. **(B)** Heatmap showing relative abundances for main microbes potentially producing various enzymes. Color bars represent the row-scaled value, in which a blue curve illustrates the distribution density percentage.

Microbial community composition can shape enzyme patterns (Rietl et al., 2016). The bacterial community structure of seven stations (Figure 8) was investigated herein. Many marine bacterial phyla such as Bacteroidetes, Planctomycetes, Chloroflexi and Proteobacteria, which have the ability to produce a variety of extracellular enzymes, particularly polysaccharases (Teske et al., 2011), were found and most of them were abundant in this study. In the 16S rRNA gene clone libraries, marine *Roseobacter* lineages (OTU_3) within the class Alphaproteobacteria dominated. They are common members of coastal bacterioplankton and are often observed as particle-colonizers (Teske et al., 2011). The next most abundant lineage was *Pseudoalteromonas* (OTU_8) within the class Gammaproteobacteria. This lineage can produce a broad range of hydrolases (e.g., alginate lyase, carrageenase and peptidase) in response to available phytoplankton detritus (Gihring et al., 2009; Qin et al., 2010; Xu et al., 2017). Other gammaproteobacterial genera (*Alteromonas* (OTU_5), *Vibrio* (OTU_149), *Pseudomonas* (OTU_91), *Psychrobacter* (OTU_614) and *Shewanella* (OTU_239)) that can secrete various enzymes to hydrolyze Tween 60, pullulan and alginate were also found (Groudieva et al., 2004; Teske et al., 2011). Cultivating and molecular assays showed that some Chloroflexi populations can secrete xylanase, amylase, chitinase, esterase, galactosidase, and glucuronidase (Kragelund et al., 2007). Sphingobacteriia, Flavobacteriia, Cytophagia and Bacteroidia within the phylum Bacteroidetes were observed in this study. They are candidates for the hydrolysis of complex HMW carbohydrates and could assimilate phytoplankton phytodetritus quickly (Gihring et al., 2009).

In this study, a variety of high enzyme activities were potentially coupled with the bacterial clades Bacteroidetes, *Roseobacter*, *Alteromonas*, and *Pseudoalteromonas*, as also reported in a mesocosm study (Allison et al., 2012). However, these marine enzyme-producing bacteria were mostly selectively identified by whether they could be cultured or by whole-genome analyses, and these taxa were assumed to be responsible for the extracellular enzymes used in the *in situ* organic matter degradation. Next, together with *in situ* metatranscriptomic and metaproteomic analyses (Bergauer et al., 2018; Orsi et al., 2018), investigation on the direct links between different enzymes and microbial communities is needed.

## Conclusion

This study investigated the distribution patterns of EEA in Chinese marginal seas. The enzyme activity showed geographical distribution pattern with latitude, which can mostly be explained by variations in DOC content. NAGase, BGase and Pase activities from inshore stations were mainly associated with chl-a, salinity and PO_4_^3–^ due to high freshwater input. High temperatures might also promote increased enzyme activity. Tested substrates were hydrolyzed at different rates and Pase, BGase and casease contributed the most to the summed activities. For each enzyme activity, the contribution of dissolved to total EEA was strongly variable between stations. Despite having the same chemical structures, differences in enzyme activity of tested polymers and their corresponding oligomers were intricate, which suggested that molecule size does not necessarily limit the turnover of marine organic matter. In addition, high enzyme activities were potentially coupled with the bacterial clades Bacteroidetes, Planctomycetes, Chloroflexi, *Roseobacter*, *Alteromonas*, and *Pseudoalteromonas*. In the future, more efforts will be needed to investigate the direct links between different enzyme activities and enzyme-producing communities.

## Data Availability

The datasets generated for this study can be found in the National Center for Biotechnology Information (NCBI) Short Read Archive database: PRJNA517539.

## Author Contributions

YL, L-LS, Y-YS, and Q-QC performed the laboratory work. YL, L-LS, and C-YL collected the samples. YL wrote the manuscript. Q-LQ helped in the data analysis. Q-LQ, C-YL, X-LC, X-YS, MW, AM, and D-LZ helped to revise the manuscript. Q-LQ and Y-ZZ designed the research.

## Conflict of Interest Statement

The authors declare that the research was conducted in the absence of any commercial or financial relationships that could be construed as a potential conflict of interest.
