# Supplementary material for: Extracellular Enzyme Activity and Its Implications for Organic Matter Cycling in Northern Chinese Marginal Seas
Source: Front Microbiol. 2019 Sep 13;10:2137. doi: 10.3389/fmicb.2019.02137 (PMC6755343; doi:10.3389/fmicb.2019.02137)
Supplement: Supplementary file 1 [file Data_Sheet_1.docx]

***Supplementary Data***

**Extracellular enzyme activity and its implications for organic matter cycling in northern Chinese marginal seas**

Yi Li^1^, Lin-Lin Sun^1^, Yuan-Yuan Sun^1^, Qian-Qian Cha^1^, Chun-Yang Li^2^, Dian-Li Zhao^3^, Xiao-Yan Song^1^, Min Wang^2^, Andrew McMinn^2,4^ Xiu-Lan Chen^1^, Yu-Zhong Zhang^1,2,3^, Qi-Long Qin^1^*

^1^State Key Laboratory of Microbial Technology, Marine Biotechnology Research Center, Shandong University, Qingdao 266237, China

^2^College of Marine Life Sciences, Ocean University of China, Qingdao 266003, China

^3^Laboratory for Marine Biology and Biotechnology, Qingdao National Laboratory for Marine Science and Technology, Qingdao, China

^4^Institute for Marine and Antarctic Studies, University of Tasmania, Hobart, Tasmania, Australia

* Correspondence should be addressed to Qin-Long Qin

E-mail: qinqilong@sdu.edu.cn

**Supplementary Figures and Tables**

**Supplementary Figure**


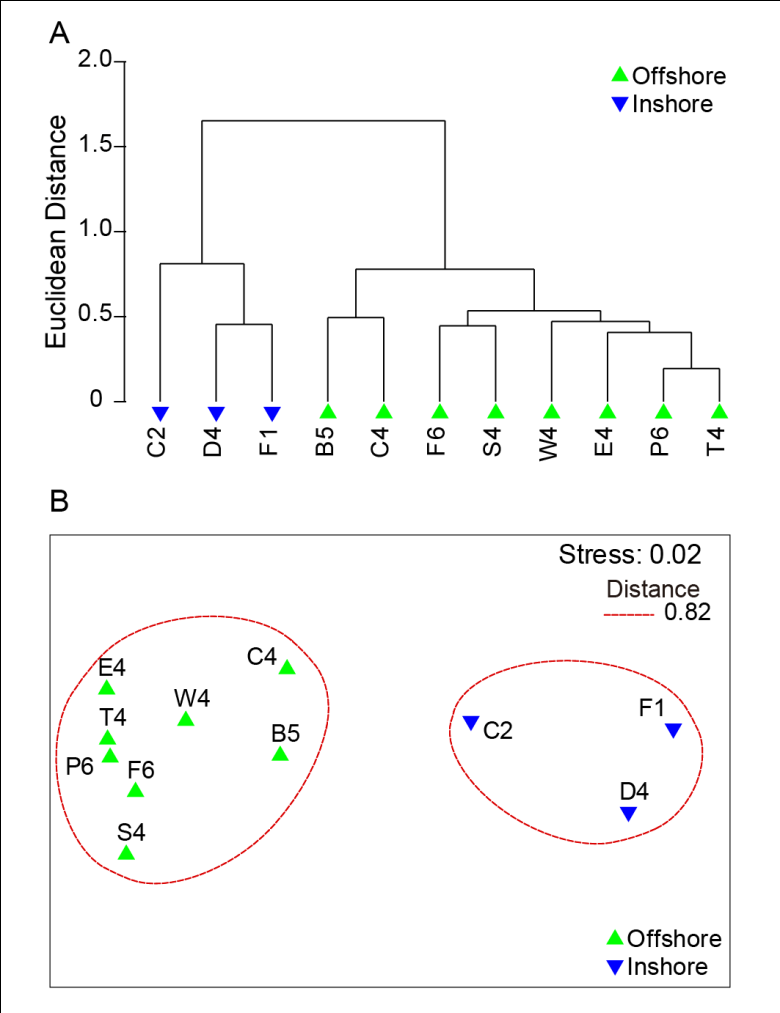


**Figure S1.** The ordination of tested environmental parameters among stations based on Euclidean distance. (A) The cluster dendrogram. (B) The nonmetric multidimensional scaling (NMDS). All stations were divided into two groups: Inshore and Offshore.

**
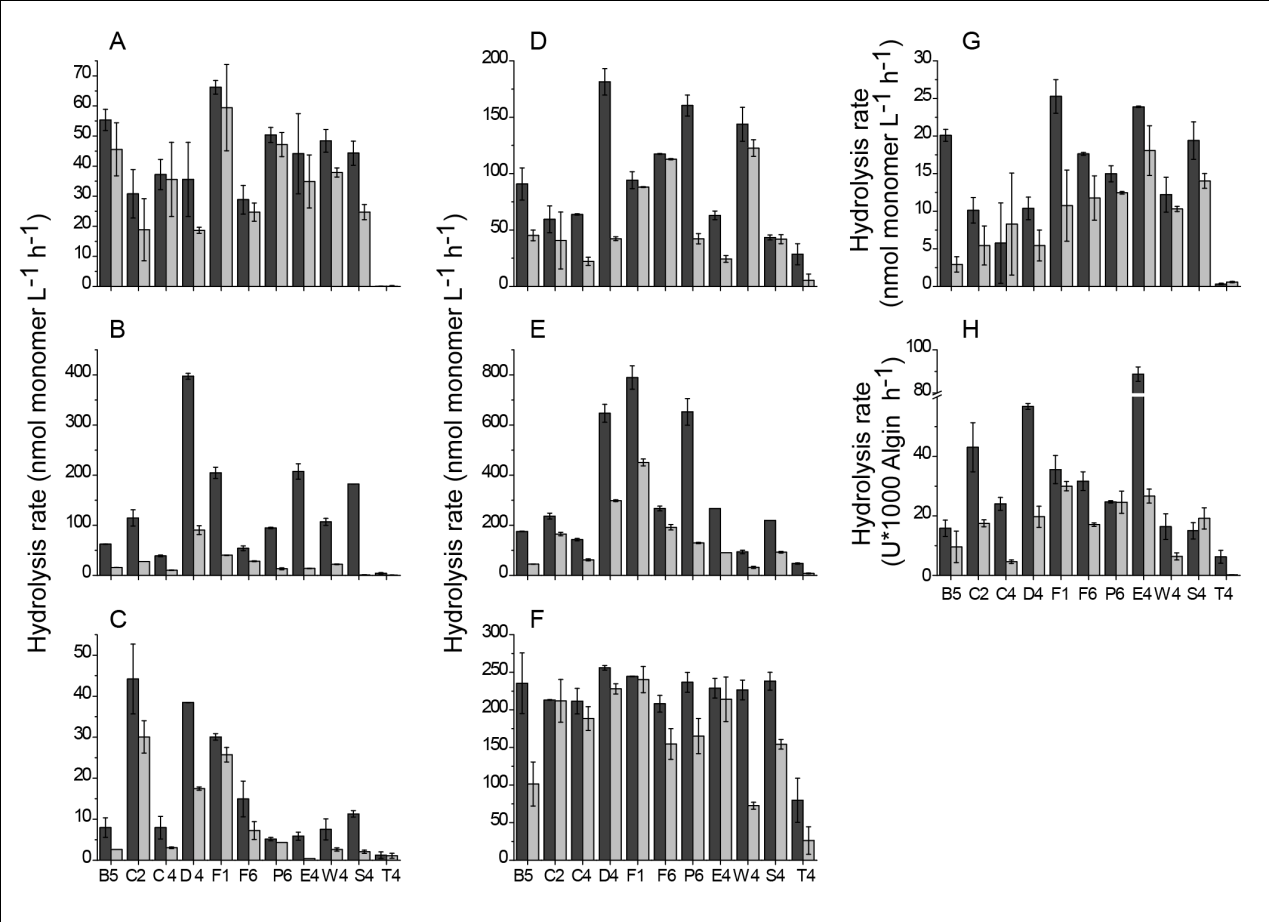
**

**Figure S2.** The hydrolysis rates of tested substrates at 25°C. (A) Carboxymethyl cellulose (CMC). (B) β-D-glucopyranoside (BG). (C) N-acetyl-β-D-glucopyranoside (NAG). (D) Laurate (C12). (E) Phosphate disodium salt hexahydrate (PDSH). (F) Casein. (G) Chitin. (H) Alginic acid sodium salt (AASS). Values are the mean hydrolysis rate in unfiltered water (dark gray), and 0.22-μm filtered water (light gray) at each station.

**
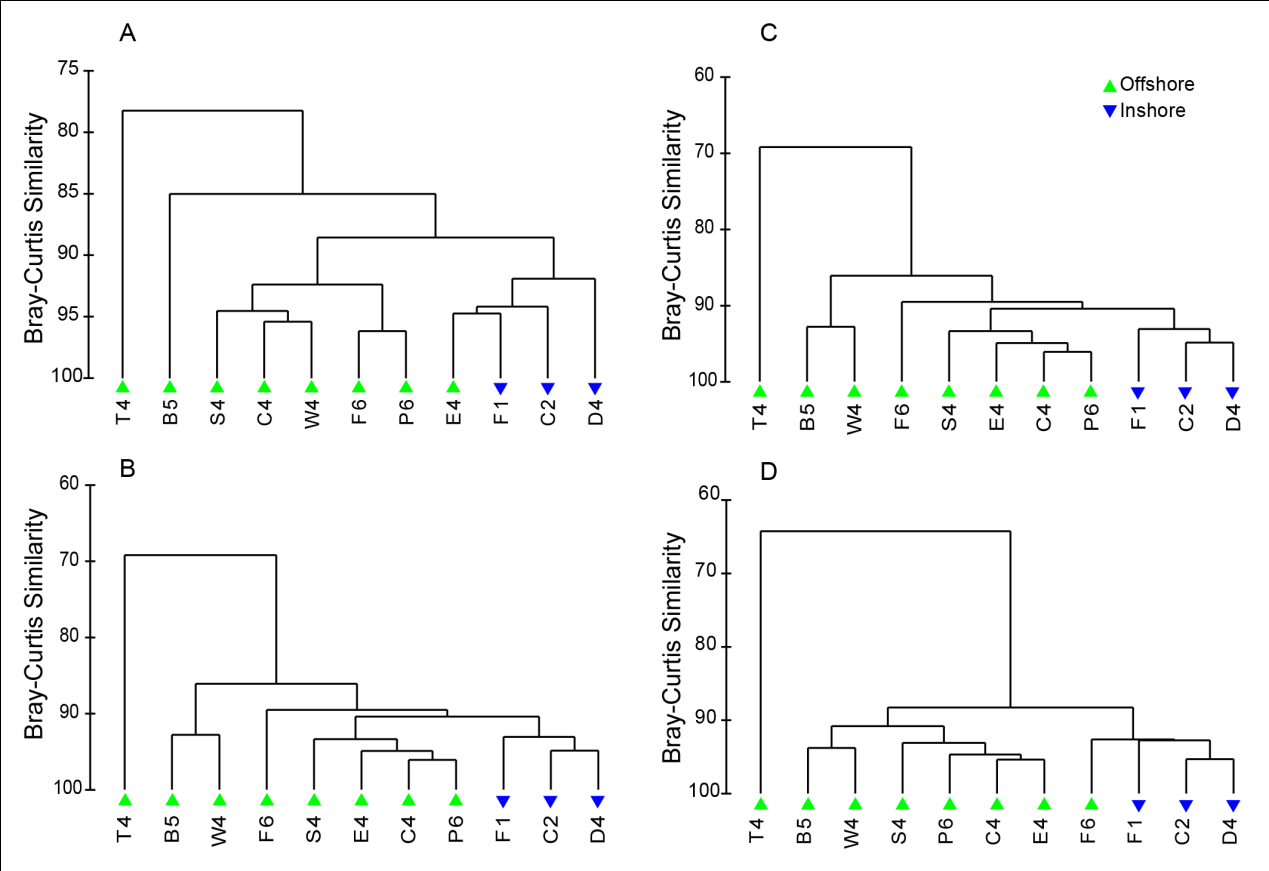
**

**Figure S3**. The cluster dendrogram of tested extracellular enzyme activities among stations based on Bray-Curtis similarity. (A) The total enzyme activities at 35°C. (B) The dissolved enzyme activities at 35°C. (C) The total enzyme activities at 25°C. (D) The dissolved enzyme activities at 25°C. All stations were divided into two groups: Inshore and Offshore.

**
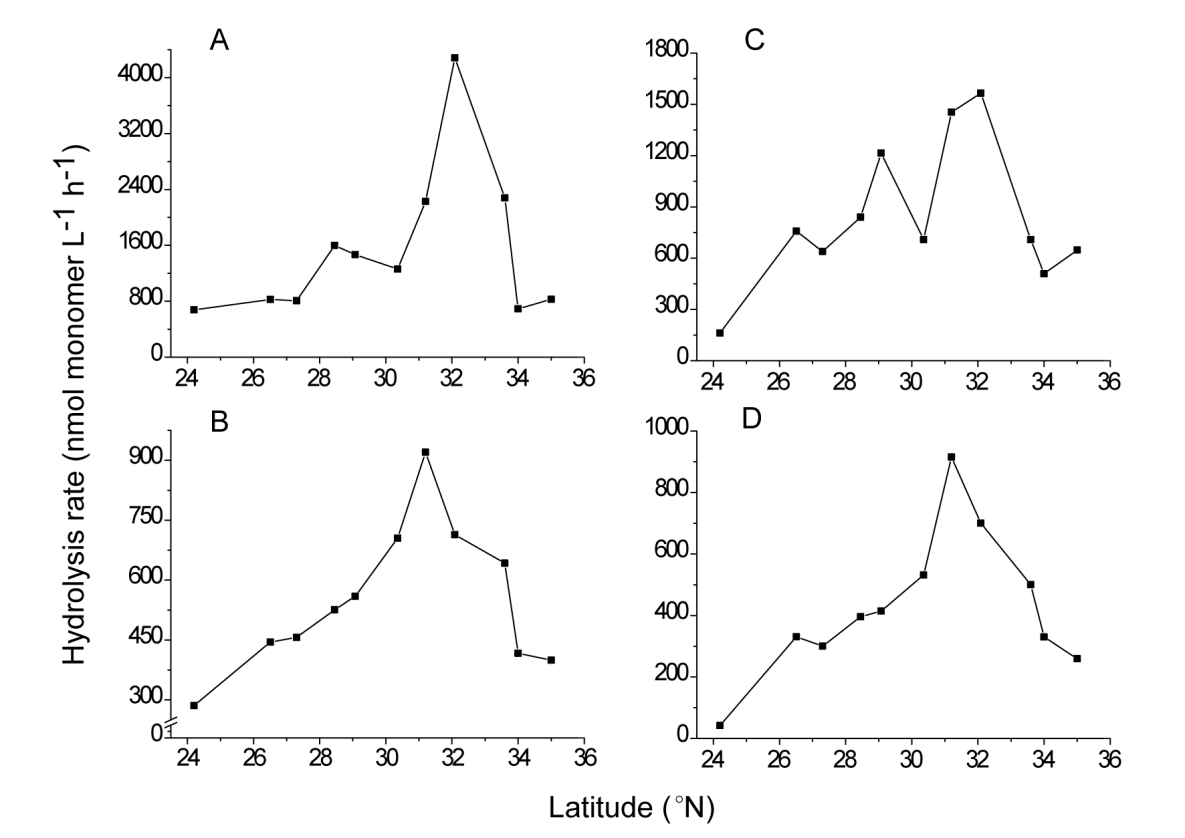
**

**Figure S4.** The changes of summed hydrolysis rates for all tested substrates along latitude. (A) The unfiltered waters at 35°C. (B) The 0.22-μm filtered waters at 35°C. (C) The unfiltered waters at 25°C. (D) The 0.22-μm filtered waters at 25°C.

**Supplementary Tables**

Table S1. Environmental variables of all the seawater samplings.

| Station | Latitude (°N) | longitude (°E) | Salinity (PSU) | Temperature (°C) | DOC  (µmol C/L) | DO (mg/dm3) | Chl-a (μg/L) | NH_4_^+^ (μmol/L) | PO_4_^3-^ (μmol/L) | NO^3-^ (μmol/L) | NO^2-^ (μmol/L) |
| --- | --- | --- | --- | --- | --- | --- | --- | --- | --- | --- | --- |
| C2 | 33.60 | 122.09 | 32.18 | 19.18 | 101.67 | 7.95 | 1.99 | 0.27 | 0.07 | 1.2 | 0.22 |
| D4 | 32.09 | 123.30 | 26.85 | 25.84 | 111.00 | 8.44 | 1.22 | 0.17 | 0.15 | 8.4 | 0.78 |
| F1 | 31.20 | 122.58 | 29.36 | 24.79 | 95.83 | 6.09 | 3.79 | 0.51 | 0.09 | 10.0 | 0.84 |
| B5 | 35.00 | 123.58 | 32.19 | 22.35 | 104.00 | 7.67 | 0.38 | 0.13 | 0.02 | 0.1 | 0.01 |
| C4 | 34.00 | 123.58 | 31.03 | 23.03 | N.A. | 8.35 | 0.16 | 0.07 | 0.06 | 0.4 | N.D. |
| E4 | 28.45 | 123.56 | 32.01 | 28.17 | 79.28 | 6.92 | 1.09 | 0.06 | 0.02 | N.D. | N.D. |
| F6 | 30.36 | 125.60 | 31.93 | 25.38 | 96.58 | 7.25 | 0.21 | 0.04 | 0.01 | N.D. | 0.02 |
| P6 | 29.07 | 125.30 | 33.58 | 27.45 | 81.39 | 6.79 | 0.19 | 0.01 | 0.02 | N.D. | N.D. |
| S4 | 26.51 | 122.12 | 34.14 | 28.00 | 88.17 | 6.61 | 0.16 | N.D. | 0.03 | N.D. | 0.03 |
| T4 | 24.20 | 119.15 | 34.17 | 25.67 | 71.61 | N.A. | 0.21 | 0.02 | 0.04 | N.D. | N.D. |
| W4 | 27.30 | 123.21 | 33.81 | 28.07 | 86.42 | 6.43 | 0.18 | 0.01 | 0.02 | 0.0 | N.D. |

N.A., no analysis; N.D., non detectable.

Table S2. Average hydrolysis rates of the polymers and corresponding monomers in inshore and offshore stations (nmol L^-1^ h^-1^).

| Temperature (°C) | water | Shore | NAG | Chitin | BG | CMC |
| --- | --- | --- | --- | --- | --- | --- |
| 35 | Unfiltered | Offshore | 3.58 | 11.55 | 37.70 | 32.93 |
|  |  | Inshore | 48.68 | 9.95 | 145.27 | 33.12 |
|  | 0.22-μm filtered | Offshore | 11.43 | 16.52 | 186.86 | 46.06 |
|  |  | Inshore | 89.56 | 15.75 | 608.73 | 55.22 |
| 25 | Unfiltered | Offshore | 2.93 | 11.11 | 13.16 | 35.78 |
|  |  | Inshore | 24.42 | 7.21 | 52.84 | 32.31 |
|  | 0.22-μm filtered | Offshore | 7.74 | 16.27 | 93.81 | 44.07 |
|  |  | Inshore | 37.58 | 15.25 | 238.87 | 44.18 |
